# Draft Genome Sequence of the Redox-Active Enteric Bacterium Citrobacter portucalensis Strain MBL

**DOI:** 10.1128/MRA.00695-20

**Published:** 2020-08-06

**Authors:** Lev M. Tsypin, Scott H. Saunders, Yinon Bar-On, Jared R. Leadbetter, Dianne K. Newman

**Affiliations:** a Division of Biology and Biological Engineering, California Institute of Technology, Pasadena, California, USA; b Department of Plant and Environmental Sciences, Weizmann Institute of Science, Rehovot, Israel; c Division of Engineering and Applied Science, California Institute of Technology, Pasadena, California, USA; d Division of Geological and Planetary Sciences, California Institute of Technology, Pasadena, California, USA; University of Southern California

## Abstract

We grew a soil enrichment culture to identify organisms that anaerobically oxidize phenazine-1-carboxylic acid. A strain of Citrobacter portucalensis was isolated from this enrichment and sequenced by both Illumina and PacBio technologies. It has a genome with a length of 5.3 Mb, a G+C content of 51.8%, and at least one plasmid.

## ANNOUNCEMENT

During the Microbial Diversity 2017 program at the Marine Biological Laboratory (MBL), we aimed to isolate organisms that anaerobically oxidize phenazine-1-carboxylic acid (PCA), a process that has never been described. Topsoil samples from Falmouth, Massachusetts (41°36'58.9"N, 70°34'31.2"W; 41°32'42.8"N, 70°37'52.4"W; and 41°31'34.3"N, 70°39'05.3"W), were incubated anoxically in Balch tubes with a minimal medium containing acetate as a nonfermentable carbon source, reduced PCA and acetate as electron donors, and nitrate as the terminal electron acceptor (https://doi.org/10.17504/protocols.io.bh4tj8wn). PCA-oxidizing enrichments were serially passaged. One strain was isolated based on its ability to anaerobically oxidize PCA and sequenced. After isolation and for sequencing, it was grown in LB medium, Miller (product number 244620; BD Difco), at 30°C with shaking at 250 rpm. It was stored as 35% glycerol stocks in a −80°C freezer.

For Illumina sequencing, DNA was extracted using the DNeasy blood and tissue kit (product number 69504; Qiagen). The library was prepared using a NEBNext kit (product number E7335; New England Biolabs) and sequenced to 100× coverage (5 million 100-bp single-end reads) on a HiSeq 2500 instrument. Base calls were performed with RTA v1.13.48.0, followed by conversion to fastq files with bcl2fastq v1.8.4. The reads were concatenated into a single file, trimmed with Trimmomatic v0.39 (with the following parameters: leading, 27; trailing, 27; slidingwindow, 4:20; minlen, 80) (1), and analyzed for quality using FastQC v0.11.8 (http://www.bioinformatics.babraham.ac.uk/projects/fastqc). For PacBio sequencing, DNA was extracted with phenol-chloroform. The library was prepared using the SMRTbell Express template preparation kit v2.0 with barcoded overhang adapters and was sequenced in a multiplexed PacBio Sequel II single-molecule real-time (SMRT) cell. This yielded 4,335 reads with a mean length of 9,840 bases for approximately 8× coverage, with an *N*_50_ value of 103,465 nucleotides (nt) given by Canu v2.0 (set genomesize, 5.3m; mininputcoverage, 7; stoponlowcoverage, 7) (2, 3). The PacBio fastq and trimmed Illumina reads were coassembled using SPAdes v1.13.1 (4). The resulting scaffolds were further improved by comparison to nine reference *Citrobacter* genomes using MeDuSa v1.6 (Table 1) (5). Contaminant sequences from eukaryotes were identified using NCBI BLAST and removed from the genome, along with scaffolds shorter than 200 nt, and the remaining scaffolds were analyzed with QUAST v5.0.2 (6). Default parameters were used for all software unless otherwise specified.

**TABLE 1:** Whole-genome comparisons of *C. portucalensis* MBL to references

Reference genome (GenBank accession no.)	BLAST ANI (%)	Proportion BLAST aligned (%)	MUMmer ANI (%)	Proportion MUMmer aligned (%)	Pearson's correlation coefficient for tetranucleotide usage
*C. portucalensis* A60^T^ (MVFY00000000.1)	98.42^*a*^	84.79	98.78^*a*^	85.43	0.99923^*a*^
*C. portucalensis* Effluent_1 (NZ_CP039327.1)	97.81^*a*^	82.92	98.31^*a*^	83.43	0.99908^*a*^
*Citrobacter braakii* FDAARGOS_253 (NZ_CP020448.2)	92.5	79.75	93.15	80.66	0.99707^*b*^
*Citrobacter werkmanii* BF-6 (NZ_CP019986.1)	90.32	78.4	91.15	78.6	0.9963^*b*^
*Citrobacter freundii* CFNIH1 (NZ_CP007557.1)	90.32	77.25	91.1	77.87	0.99596^*b*^
*Citrobacter youngae* NCTC13709 (NZ_LR134485.1)	89.27	75.07	90.33	74.52	0.99759^*b*^
*Citrobacter koseri* ATCC BAA-895 (NC_009792.1)	83.05	65.21	85.43	54.95	0.97119
*Citrobacter amalonaticus* Y19 (NZ_CP011132.1)	81.74	66.53	85.23	49.16	0.98547
*Citrobacter farmeri* AUSMDU00008141 (NZ_CP022695.1)	81.47	66.96	85.01	49.17	0.98683
*Citrobacter rodentium* ICC168 (NC_013716.1)	81.28	62.37	84.98	43.83	0.94412
*E. coli* O157:H7 Sakai (NC_002695.2)	80.28	62.16	84.67	38.66	0.97933

a Values above the threshold for species identity (7).

b Values within the range for species identity (7).

**FIG 1:**
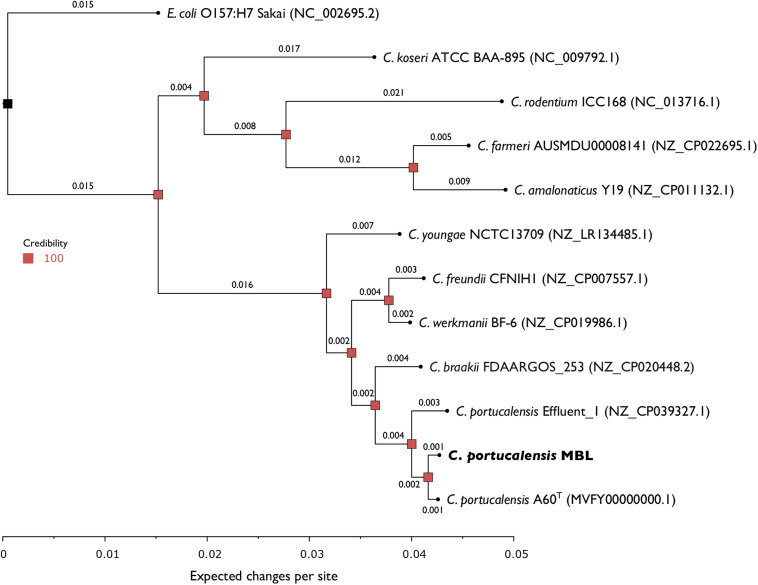
MLSA phylogeny of the genomes compared in Table 1. C. portucalensis MBL is shown in bold at the bottom of the tree. The E. coli strain is the outgroup and roots the tree. This phylogeny was calculated based on the alignment of concatenated HMM profiles of 32 single-copy nonribosomal housekeeping genes that are shared across all 12 genomes (PFAM accession numbers PF00709.21, PF00406.22, PF01808.18, PF00231.19, PF00119.20, PF01264.21, PF00889.19, PF01176.19, PF02601.15, PF01025.19, PF01725.16, PF01715.17, PF00213.18, PF01195.19, PF00162.19, PF02033.18, PF02565.15, PF00825.18, PF01193.24, PF01192.22, PF01765.19, PF02410.15, PF03652.15, PF00584.20, PF03840.14, PF00344.20, PF01668.18, PF00750.19, PF01746.21, PF02367.17, PF02130.17, and PF02699.15).

We identified the isolate as a Citrobacter portucalensis strain (which we designated strain MBL) by average nucleotide identity (ANI) and tetranucleotide usage correlations using JSpeciesWS (Table 1), and we validated this finding using multilocus sequence analysis (MLSA) (Fig. 1) (7). MLSA was performed using anvi'o v6.1 to generate hidden Markov model (HMM) profiles for 32 nonribosomal single-copy housekeeping genes common to all reference strains compared (Fig. 1) (8). These HMM profiles were aligned using MUSCLE, and a phylogeny was constructed with MrBayes v3.2.7a on the CIPRES Science Gateway, with the reference Escherichia coli strain as the outgroup (Fig. 1) (9–11). Both the MLSA and whole-genome comparisons returned the type strain C. portucalensis A60^T^ as the closest relative to C. portucalensis MBL. In the whole-genome comparisons, only C. portucalensis Effluent_1 and A60^T^ gave values above the thresholds for species identity (7, 12–16). The C. portucalensis MBL genome has a total length of 5,311,497 nt with seven total scaffolds, none of which is circularized. The *N*_50_ value is 5,245,291 nt and corresponds to the single chromosome scaffold. There are two putative plasmid scaffolds, which we named pCpMBL1 and pCpMBL2 (50,894 bp and 5,198 bp, respectively) and which we identified by homology; pCpMBL1 is likely an F plasmid and contains homologs to all components of the conjugation apparatus. We annotated the genome using the Prokaryotic Genome Annotation Pipeline (PGAP) (17, 18). Current research on C. portucalensis MBL is directed toward understanding its redox physiology.

### Data availability.

This genome has been deposited at DDBJ/ENA/GenBank under the accession number JABVAY000000000. The version described in this paper is version JABVAY010000000. The genome and raw reads are associated with BioProject PRJNA638116. The SRA accession number for the Illumina reads is SRR11952884, and the SRA accession number for the PacBio reads is SRR11952883.
